# Evidence for loss of synaptic AMPA receptors in anterior piriform cortex of aged mice

**DOI:** 10.3389/fnagi.2013.00039

**Published:** 2013-08-06

**Authors:** James Gocel, John Larson

**Affiliations:** Psychiatric Institute (M/C 912), Department of Psychiatry, College of Medicine, University of Illinois at ChicagoChicago, IL, USA

**Keywords:** piriform cortex, mEPSC, LTP, AMPA, aging, mouse, olfaction

## Abstract

It has been suggested that age-related impairments in learning and memory may be due to age-related deficits in long-term potentiation of glutamatergic synaptic transmission. For example, olfactory discrimination learning is significantly affected by aging in mice and this may be due, in part, to diminished synaptic plasticity in piriform cortex. In the present study, we tested for alterations in electrophysiological properties and synaptic transmission in this simple cortical network. Whole-cell recordings were made from principal neurons in slices of anterior piriform cortex from young (3–6 months old) and old (24–28 months) C57Bl/6 mice. Miniature excitatory postsynaptic currents (mEPSCs) mediated by AMPA receptors were collected from cells in presence of tetrodotoxin (TTX) and held at -80 mV in voltage-clamp. Amplitudes of mEPSCs were significantly reduced in aged mice, suggesting that synaptic AMPA receptor expression is decreased during aging. In a second set of experiments, spontaneous excitatory postsynaptic currents (s/mEPSCs) were recorded in slices from different cohorts of young and old mice, in the absence of TTX. These currents resembled mEPSCs and were similarly reduced in amplitude in old mice. The results represent the first electrophysiological evidence for age-related declines in glutamatergic synaptic function in the mammalian olfactory system.

## INTRODUCTION

Changes in the nervous system with aging are profound and mysterious. The brain exhibits subtle alterations in cellular morphology, synaptic structure, gene expression patterns, and electrophysiological characteristics as it ages; less subtle, perhaps, are the sensory, motor, and cognitive declines that accompany the aging process. Understanding the relationships between neurobiological changes and functional outcomes is one of the fundamental challenges of aging neuroscience. Considerable progress has been made in correlating age-dependent changes in hippocampal circuitry to spatial learning and memory deficits in aging animals. Two general principles have emerged from studies of hippocampal long-term potentiation (LTP) in aged animals (Burke and Barnes, 2010): first, age-dependent effects on synaptic function are regionally heterogeneous. For example, synaptic density declines in the dentate gyrus, but not field CA1, of old rats. Electrophysiological studies using minimal stimulation suggest that “basal” synaptic potency, the average size of the postsynaptic response to a presynaptic release event (Stevens and Wang, 1994), declines with extreme age in CA1 but not dentate. Second, LTP induction mechanisms are typically only impaired when stimulation is close to the induction threshold; LTP expression, provided that induction conditions are suprathreshold, appears normal (Burke and Barnes, 2010).

The olfactory system has a number of advantages for neurobiological studies of sensory and cognitive functions in aging. Olfactory discrimination ability declines markedly in human aging (Doty et al., 1984; Cain and Stevens, 1989) and appears to be particularly vulnerable to age-related neurodegenerative disease (Serby et al., 1985; Kesslak et al., 1988). Olfactory dysfunction has also been reported for aging rodents (Roman et al., 1996; Frick et al., 2000; Schoenbaum et al., 2002; Enwere et al., 2004; Prediger et al., 2005; LaSarge et al., 2007). For instance, a recent study from our laboratory found that old mice took more trials to learn two-odor discrimination problems for positive reinforcement and failed to show improvement across multiple discrimination problems when compared to young mice (Patel and Larson, 2009). However, the neurobiological bases for these deficits are largely unexplored.

The primary olfactory (piriform) cortex receives monosynaptic input from the mitral (and tufted) cells of the olfactory bulb, which themselves receive monosynaptic input from primary sensory neurons in the olfactory epithelium. Layer II pyramidal neurons of piriform cortex appear to be situated to form combinatorial representations of the different olfactory bulb glomeruli that respond to distinct molecular features of the chemicals comprising an odor (Wilson and Sullivan, 2011). Piriform neurons project monosynaptically to the lateral entorhinal cortex, providing olfactory input to the hippocampal formation, as well as to parts of the amygdalar complex and the prefrontal cortex (Shipley et al., 1995). Both the afferent synapses made by the mitral cells onto layer II pyramidal cells and the associational feedback system generated by neighboring pyramidal cells are glutamatergic and exhibit LTP (Jung et al., 1990a,b; Kanter and Haberly, 1990), possibly to strengthen representations of learned odors or to participate in odor–reward associations. The present studies were directed to test for synaptic functional changes in anterior piriform cortex (APC) of aged mice. The results provide the first direct evidence that aging results in decreases in synaptic currents mediated by AMPA-type glutamate receptors in a simple cortical network.

## MATERIALS AND METHODS

### ANIMALS

C57Bl/6J mice were bred in the Psychiatric Institute vivarium from breeding stock obtained from Jackson Laboratories (Bar Harbor, ME, USA). Mice were weaned at 21 days of age and males were separated and housed in groups of two to four per cage until sacrificed for experiments. Food and water were available *ad libitum* and routine veterinary visits ensured that animals were in good health prior to experimentation. Any animals that displayed physical ailments or lethargy were excluded from this study. Electrophysiological experiments were conducted on brain slices obtained from mice aged 3–6 months (“young”; *n* = 16) or 24–28 months (“old”; *n* = 12). All procedures were in accordance with NIH guidelines and protocols were approved by the Animal Care Committee of the University of Illinois at Chicago (UIC ACC #09-232).

### ELECTROPHYSIOLOGY

#### In vitro slice preparation and electrophysiology

Parasagittal slices (300 μm) of APC were prepared in the usual method. Mice were decapitated and brains removed in oxygenated artificial cerebral spinal fluid (aCSF) containing (in mM): NaCl (120), KCl (3.1), K_2_HPO_4_ (1.25), NaHCO_3_ (26), dextrose (5.0), L-ascorbate (2), MgCl_2_ (1.0), CaCl_2_ (2.0) at ~4°C. The brain was then sectioned into blocks (2–3 mm thick), mounted on a cutting stage, and sliced on a vibrating cutter (Vibratome, St. Louis, MO, USA). The slices were incubated at 32°C for 1 h and then allowed to cool to room temperature (~25–27°C). Slices were then transferred to a submerged recording chamber and perfused with aCSF. Recordings were obtained from principal cells in layer II at room temperature, as described previously (Gocel and Larson, 2012). Patch electrodes (1.8–3 MΩmega) contained (in mM): cesium methanesulfonate (145), MgCl_2_ (1), HEPES (10), BAPTA (1.1), MgATP (5), and phosphocreatine (20) adjusted to pH 7.2 with CsOH, 290 mOsm. Cells were visualized with differential interference contrast (DIC) optics on a Nikon Eclipse E600FN “PhysioStation” (Nikon, Melville, NY, USA). Twisted bipolar electrodes (custom made) with a tip diameter ~50 μm were positioned in layer Ia and Ib 150–200 μm rostral from the recorded cell. Stimulation (0.1 ms pulses 1–200 μA) in these layers activate afferent lateral olfactory tract (LOT) and intrinsic association (ASSN) fibers synapsing onto principal cells in layer II. Responses at ASSN synapses display paired-pulse depression whereas stimulation of afferent LOT fibers in layer Ia exhibit facilitation (Bower and Haberly, 1986). Therefore, stimulation of ASSN fibers in layer Ib was confirmed after obtaining whole-cell recording by non-facilitating responses to paired-pulse stimulation at a 200 ms inter-pulse interval (IPI). Evoked excitatory postsynaptic currents (EPSCs), miniature EPSCs (mEPSCs) and spontaneous EPSCs (s/mEPSCs) were recorded with an Axopatch-1D amplifier and pClamp software (Molecular Devices, Sunnyvale, CA, USA), filtered at 1 kHz, digitized at 10 kHz, and stored on the computer hard drive. Cells were immediately rejected if series resistance exceeded 15 MΩmega upon obtaining whole-cell recording configuration. Series and whole-cell capacitance compensation and junction potential correction were not used.

### PHARMACOLOGY

All drugs and chemicals were applied via the perfusate by a solenoid-controlled gravity-feed system (ValveLink 8, AutoMate Scientific, Inc., Berkeley, CA, USA). The rate of flow of all drug perfusates was equilibrated to 2 mL/min prior to the inception of experimentation. GABA_A_ mediated transmission was blocked by 25 μM 1(S),9(R)-(-)-bicuculline methiodide (BMI) in all experiments in order to isolate postsynaptic excitatory currents. The NMDA receptor antagonist, 3-[(R)-2-carboxypiperazin-4-yl]-prop-2-enyl-1-phosphonic acid (CPP, 20 μM)) was used to isolate AMPA receptor-mediated currents. 1 μM tetrodotoxin (TTX) was added to the perfusate in order to eliminate spontaneous action potential-dependent events. Some experiments used 6-cyano-7-nitroquinoxaline-2,3-dione (CNQX) to block AMPA receptors (20 μM). All drugs were taken from stock solutions dissolved in H_2_O and diluted in aCSF as needed.

### EVOKED SYNAPTIC CURRENTS

In experiments using synaptic stimulation under voltage-clamp, the following stimulation protocol was used: each trial began with a baseline recording period of 300 ms, followed by paired-pulse stimulation of the LOT (50 ms IPI), a one-second delay, and paired-pulse stimulation of the ASSN fibers (50 ms IPI). Stimulation current levels were set to yield a reliable response with the least amount of stimulation in order to avoid polysynaptic activity or synaptic population crosstalk. Stimulation intensities were the following: LOT: 3–6 months, 40.61 ± 12.12 μA; 24–28 months, 120.00 ± 47.97 μA and ASSN: 3–6 months, 26.17 ± 3.02 μA, 24–28 months, 60.89 ± 11.17 μA. Any recordings demonstrating polysynaptic activity were excluded from analysis. Measurements of averaged (50–100 trials) current attributes were performed in Clampfit.

### mEPSC AND s/mEPSC ANALYSIS

For analysis of mEPSCs, slices were perfused with TTX (1 μM) until whole-cell currents evoked by LOT and ASSN fiber stimulation were abolished. Cells were maintained at a holding potential of -80 mV throughout recordings and bicuculline and CPP were used to prevent activation of GABA_A_ and NMDA receptors, respectively. Spontaneous synaptic currents were recorded for 200 s of continuous recording from every cell. mEPSCs were identified as follows: A single 50 ms variable amplitude template was constructed (Clements and Bekkers, 1997) from >20 visually-identified events in a randomly-selected cell and served as a search criterion for collecting and aligning mEPSCs and s/mEPSCs for all cells. Cells were excluded from analysis if the baseline drifted more than 50 pA or if access resistance changed more than 20% throughout the 200 s of continuous recording. All compound events were excluded from analysis. Measurements of the current waveforms (kinetics) were obtained from an average of all events (>100) that met analysis criteria in each cell. Decay time constants were fit to averaged mEPSCs with the standard exponential equation *I*(*t*) = *I* × exp(-*t*/τ ), where *I* is the peak amplitude and τ is a the decay time constant.

Spontaneous EPSCs (s/mEPSCs) were recorded and quantified as for mEPSCs, except that TTX was not used.

### HISTOLOGY

Some cells used for electrophysiology were also filled with a 4% Lucifer yellow solution passively during the recordings. Brain sections were then immediately fixed in 4% paraformaldehyde overnight, whole mounted on glass slides and coverslipped. Additional physiological slices fixed in 4% paraformaldehyde were cryoprotected in 30% sucrose in PBS. These slices were then resectioned at 30 μm, processed, and stained with 0.1% cresyl violet. Filled cells were visualized under a fluorescein isothiocyanate (FITC) filter and stained slices were visualized under bright field illumination on an Axioskop 2 microscope. Images were acquired through Axiovision software (Zeiss, Thornwood, NY, USA) and edited in GNU image manipulation program (GIMP, open source).

### STATISTICS

All data are based on cells as individual sample units and are presented as means ± SEM. Statistical differences between two groups were evaluated using Student’s unpaired *t*-test. Analysis of variance was used for comparisons involving more than two groups.

## RESULTS

**FIGURE 1:**
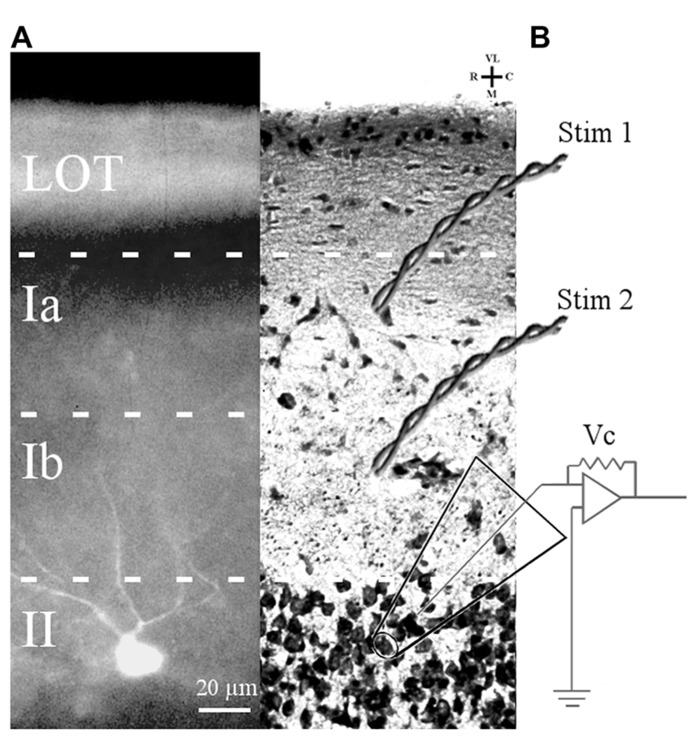
**Slice preparation of APC and arrangement of stimulation and recording electrodes.** **(A)** A Lucifer yellow-filled cell in APC slice is shown. The soma is located in layer II and primary dendrites (only partially in the focal plane) ascend through layer I. **(B)** Cresyl violet-stained APC section showing position of bipolar electrodes to stimulate LOT fibers (Stim 1) and ASSN fibers (Stim 2). Patch clamp recordings were obtained in the whole cell configuration from somata of layer II principal cells visually identified under DIC optics.

A subset of the cells chosen for electrophysiological study were filled with Lucifer yellow to confirm that they were layer II pyramidal cells (**Figure 1**). Filled cells from mice at all ages showed multiple, spiny apical dendrites which projected toward the pial surface. Other slices stained with cresyl violet were examined for cytoarchitecture. There were no obvious differences in soma morphology, cortical lamination, or cell density of APC of 3–6 months old and 24–28 months old mice.

### AMPA RECEPTOR-MEDIATED mEPSCs ARE SMALLER IN THE AGED MOUSE

**FIGURE 2:**
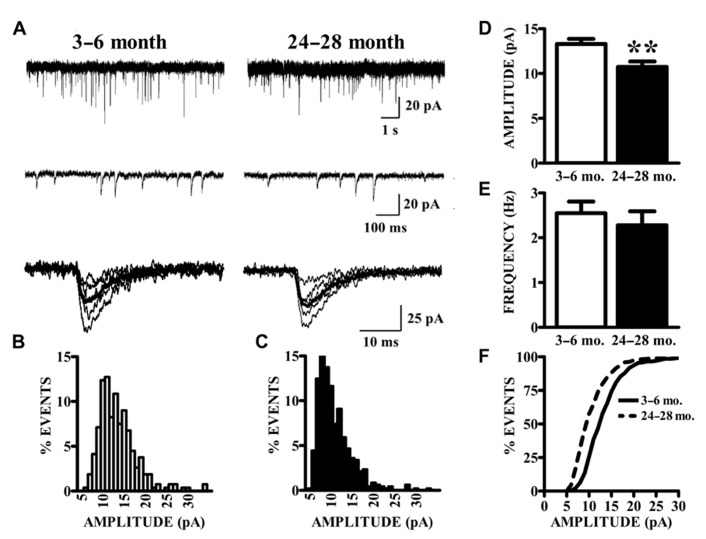
**AMPA receptor-mediated currents at single synapses are reduced in aged mice.** **(A)** mEPSCs were collected in the presence of TTX. Top raw traces were extracted from 200 s recording epochs from which spontaneous currents were collected. Middle traces are expanded sections of the top traces; a template algorithm was used to search for individual currents. Bottom traces are selected individual mEPSCs aligned according to onset of the current. **(B,C)** All currents collected during the 200 s epochs were binned according to amplitude. As demonstrated by these representative histograms, AMPA mEPSC amplitude histograms in old (24–28 months) animals **(C)** demonstrated a leftward shift in distribution as compared to young (3–6 months) animals **(B)**. **(D)** Mean amplitudes (shown) of AMPA mEPSCs were significantly decreased in old relative to young mice (*t*_36_ = 2.87, *p* < 0.01). Comparison of both age groups according to the median amplitude (not shown) also demonstrated a reduction. **(E)** The frequency of mEPSCs was comparable between age groups. Frequency was determined by counting the events that occurred during the entire 200-s recording epochs. **(F)** Cumulative distributions illustrate the shift in the mEPSC amplitudes.

As described previously (Gocel and Larson, 2012), superficial pyramidal (SP) neurons held at -80 mV under voltage clamp in the presence of BMI, CPP, and TTX showed spontaneous inward currents with the characteristics of mEPSCs, in slices from both young and old mice (**Figure 2A**). These events were abolished by perfusion with CNQX, confirming that they were mediated by AMPA receptors (data not shown). Synaptic events were collected from continuous 200 s recording epochs, aligned, averaged, and analyzed. Amplitude distributions were positively skewed in both young (**Figure 2B**) and old mice (**Figure 2C**). The mean amplitudes of mEPSCs recorded in slices from old mice were significantly smaller than those from young mice (**Figure 2D**). Since the amplitude distributions were skewed, the median mEPSC amplitude was also calculated for each cell and compared between age groups. These measures were also significantly smaller in the aged mice (young: 12.31 ± 0.51 pA; old: 9.95 ± 0.53 pA; *p* < 0.01). The average frequency of detected mEPSCs did not significantly differ between age groups (**Figure 2E**). Cumulative amplitude distributions (**Figure 2F**) also illustrate the shift in amplitude toward smaller mEPSCs in the neurons from old mice. Possible age-related changes in the kinetics of AMPA receptors mediating mEPSCs were calculated by fitting a single exponential function to the decay phase of averaged mEPSCs in each cell. The decay time constants tended to be longer in cells from old mice, although this difference only approached statistical significance (young: 4.22 ± 0.16 ms, *n* = 24; old: 4.72 ± 0.18 ms, *n* = 14; *t*_36_ = 1.95, *p* = 0.06).

### PAIRED-PULSE RESPONSES ARE UNAFFECTED BY AGING

**FIGURE 3:**
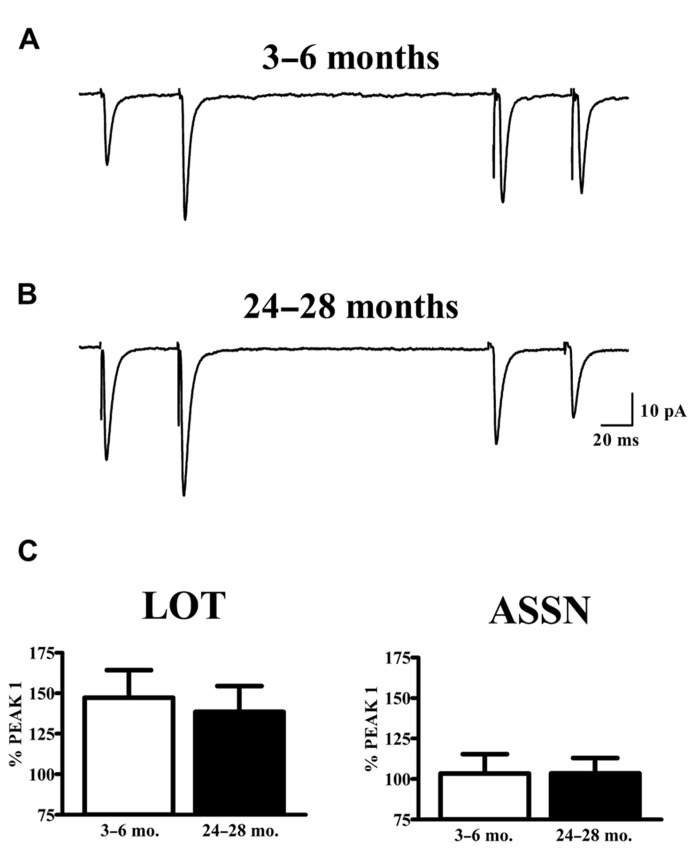
**Paired-pulse characteristics in APC at LOT and ASSN synapses are unaffected by aging.** **(A,B)** Evoked LOT (left traces) and ASSN (right traces) paired pulse responses from principal cells in layer II of APC were collected from young (3–6 months, *n* = 18) and old (24–28 months, *n* = 11) mice at –80 mV. Cells were only included in analysis if recordings were obtained from both LOT and ASSN inputs. Paired-pulse stimulation (50 ms ISI) was presented to LOT fibers followed by the same stimulation to ASSN fibers one second later. Currents were collected every 20 s and the representative traces illustrated are an average of 50–100 stimulations. **(C)** Normalized averages of the second response were generated by calculating the response amplitude relative to that of the first response. No differences in paired-pulse response at either LOT or ASSN synapses were detected between age groups.

The vast majority of glutamatergic synapses on SP neurons in APC are generated by either afferents from the olfactory bulb (LOT), terminating in the outer molecular layer (Ia) or the intrinsic associational (ASSN) system generated by the SP neurons themselves and terminating in the inner molecular layer (Ib). To test whether or not changes in the potency of individual synapses (mEPSCs) with aging were accompanied by presynaptic changes in release characteristics at either or both of these systems, paired-pulse stimulation (50 ms IPI) was applied to each pathway while recording from cells held at -80 mV in slices from young and old mice. Cells were only included in analysis if responses were present from both synaptic pathways. All traces collected for a given cell which met criteria were averaged and the percent potentiation or depression of the second response was calculated relative to that of the first response (**Figure 3**). The robust facilitation of responses to LOT stimulation was not significantly altered by age. ASSN responses showed neither facilitation nor depression at the interval tested; normalized responses were also unaffected by age.

### SPONTANEOUS SYNAPTIC CURRENTS (s/mEPSCs) ARE REDUCED IN THE AGED MOUSE

**FIGURE 4:**
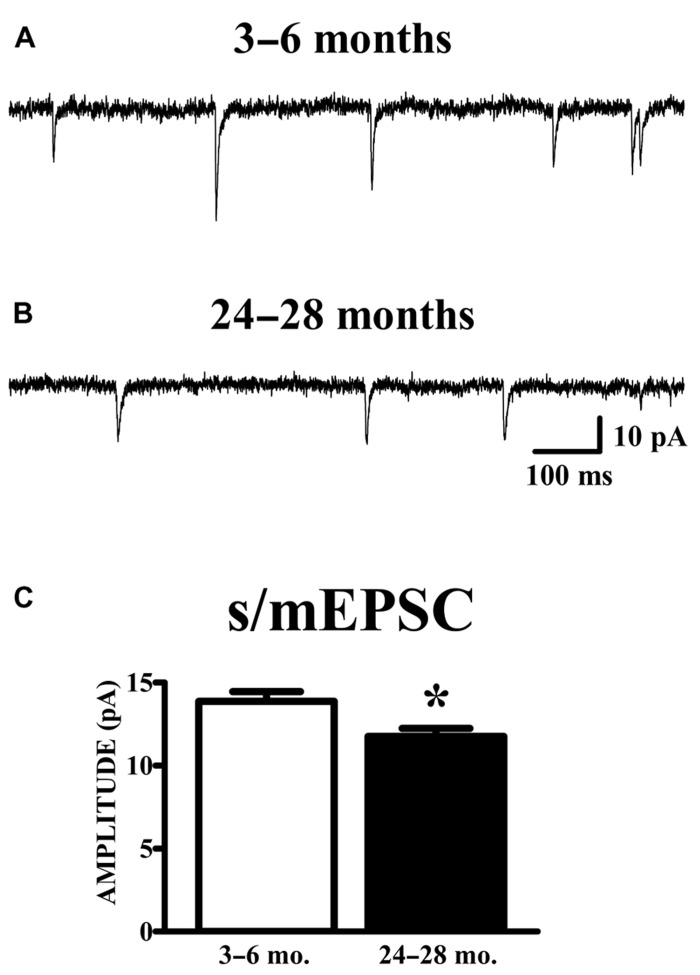
**AMPA s/mEPSC amplitudes are reduced in APC of old mice.** **(A,B)** Raw traces illustrated include events collected during recording periods where no stimulation was presented in the same cells shown in **Figure 3**. Spontaneous currents (s/mEPSCs) in these recordings are presumed to emanate from action potential-mediated and -independent events since TTX was not present in the perfusate. **(C)** The mean amplitude of s/mEPSCs was calculated and averaged across cells. Events collected from aged (24–28 months, *n* = 11) mice demonstrated a decrease in amplitude compared to those from young (3–6 months, *n* = 18) mice (*t*_27_ = 2.48, *p* < 0.05).

Spontaneous currents in the absence of stimulation were obtained from the same cells in which evoked paired-pulse responses were obtained. Experiments were performed in the absence of TTX in order to obtain evoked responses; therefore, the spontaneous currents collected are presumed to be a mixture of action potential-dependent (sEPSCs) and independent (mEPSCs) events. The manner of analysis was identical to the method of analyzing mEPSC data. Aged animals exhibited a significant reduction in mean (**Figure 4**) and median (young: 12.78 ± 0.53 pA; old: 10.69 ± 0.41 pA; *p* < 0.01) s/mEPSC amplitude. The similar size of mEPSCs recorded in the presence of TTX (**Figure 2**) and s/mEPSCs recorded in the absence of TTX (**Figure 4**) suggests either (1) that action potential-dependent and -independent release events evoke similar postsynaptic responses or (2) that the action potential-dependent (TTX-sensitive) events are a small fraction of spontaneous release events in these cells. Frequency distributions of s/mEPSC amplitudes also did not reveal any obvious differences with those of mEPSCs. In any case, it bears noting that the s/mEPSC (**Figure 4**) and mEPSC (**Figure 2**) recordings were made from completely different sets of young and old animals.

## DISCUSSION

There is compelling evidence that olfaction-mediated sensory and cognitive functions decline with aging in humans (Doty et al., 1984; Cain and Stevens, 1989; Wysocki and Gilbert, 1989; Larsson et al., 2000, 2006; Gilbert et al., 2008) and experimental animals (Roman et al., 1996; Frick et al., 2000; Enwere et al., 2004; Prediger et al., 2005; LaSarge et al., 2007; Dardou et al., 2008; Luu et al., 2008; Patel and Larson, 2009). The piriform cortex occupies a strategic position in the neural processing of odors: (i) it is the largest target of efferents from the olfactory bulb (Neville and Haberly, 2004); (ii) its internal wiring suggests a combinatorial mechanism for synthetic integration of odor percepts from multiple chemical constituents that activate distinct odorant receptor proteins and corresponding glomeruli in the olfactory bulb (Haberly, 2001; Franks and Isaacson, 2006; Stettler and Axel, 2009; Wilson and Sullivan, 2011); (iii) odor discrimination and olfactory learning are disrupted by piriform lesions (Staubli et al., 1987); (iv) olfactory training modifies synaptic structure and function in piriform cortex (Barkai and Saar, 2001); and (v) it is the primary pathway by which olfactory information reaches the hippocampus, amygdala, and prefrontal cortex (Shipley et al., 1995). However, this structure has received very little attention in neurobiological studies of aging. The present study represents an initial step in a comprehensive analysis of the brain substrate for olfactory dysfunction in aging.

We recorded from principal neurons in primary olfactory cortex from young adult and aged mice. The main finding of the experiments described here is a decrease in the amplitude of synaptic currents mediated by AMPA receptors on these cells in aged mice. TTX-resistant spontaneous synaptic currents (mEPSCs) are thought to be evoked by stochastic release of single glutamate quanta at individual synapses. Assuming that synaptic vesicles are the physical basis for quantal release, there are two main ways to alter quantal size: a change in glutamate loading of vesicles or a change in postsynaptic receptors in the synaptic zone. There is little precedent for changes in vesicle loading but considerable evidence that postsynaptic AMPA receptor numbers can be altered in an experience-dependent manner (Lynch and Baudry, 1984; Bredt and Nicoll, 2003) or in certain disease models (Li et al., 2002). Therefore the most parsimonious interpretation of a decrease in mEPSC size is a reduction in the number of functional AMPA receptors activated by synaptic glutamate release in the aged mice. In theory, a postsynaptic mechanism could be confirmed or ruled out by measuring NMDA receptor-mediated mEPSCs; however, this was impractical due to the voltage-dependence and slow kinetics of NMDA receptor-mediated currents. On the other hand, there are numerous reports of decreases in AMPA receptor expression, measured by mRNA or protein expression or ligand binding, in various brain regions of aged rodents (Bahr et al., 1992; Magnusson and Cotman, 1993; Nicoletti et al., 1995; Nicolle et al., 1996; Magnusson, 1998; Wenk and Barnes, 2000; Majdi et al., 2009).

It is important to note that spontaneous EPSCs (sEPSCs) were also recorded from APC cells in slices from completely independent cohorts of young and old mice, in the absence of TTX. The amplitude distributions of these events were almost identical to the events (mEPSCs) recorded in slices exposed to TTX. This suggests that the sEPSCs and mEPSCs are the same events; the lack of TTX sensitivity may be attributed to the high resting membrane potentials and low spontaneous firing of piriform pyramidal cells *in vitro* (Scholfield, 1978; Tseng and Haberly, 1988; Suzuki and Bekkers, 2006). In any case, the smaller amplitude of spontaneously-occurring synaptic AMPA currents in cells from old mice was replicated for both mEPSCs and “sEPSCs,” in different cohorts of mice.

The frequency of mEPSCs was not altered in the aged mice, suggesting that either (i) no change in the number of synapses contributing to these synapses with aging or (ii) that any such changes are accompanied by compensatory changes in spontaneous release probability. The synaptic origin of the mEPSCs could not be determined in these experiments. However, it is worth noting that the paired-pulse experiments did not reveal any differences that would be indicative of changes in release probability in either the LOT or ASSN pathways in young versus old mice. It is also worth noting that paired-pulse ratios may not be very sensitive measures of release kinetics.

*In toto*, the results of the present study point to the conclusion that aging results in a loss of functional AMPA receptors at synapses on layer II pyramidal cells in piriform cortex. These losses may occur at LOT synapses, ASSN synapses, or both. Further studies will be necessary to establish whether these AMPA receptor losses are due to alterations in receptor subunit transcription, translation, trafficking, post-translational modification, or receptor assembly, as well as whether or not synaptic NMDA receptors are similarly effecting by aging. The decrease in synaptic potency with aging observed in the present study is in contrast to studies using minimal stimulation in rat hippocampal formation where the results suggest that dentate synapses increase in potency (larger unitary EPSPs) while CA1 synapses remain unchanged in old age (Barnes and McNaughton, 1980; Foster et al., 1991; Barnes et al., 1992).

A loss of synaptic AMPA receptors would not be expected to be without effect on LTP in the APC. Although the effects of aging on LTP have not been studied in piriform cortex, activation of more synapses with fewer AMPA receptors would be needed to trigger LTP in aged mice, in order to overcome the voltage-dependent block of NMDA receptors during high frequency stimulation. A disturbance of LTP might not be evident with supramaximal stimulation paradigms but may appear with near threshold stimulation, as observed in hippocampal field CA1. In this scenario, enhancement of AMPA receptor function might compensate for loss of synaptic AMPA receptors. Drugs that act in this way are known to facilitate olfactory learning in young animals (Larson et al., 1995) and may alleviate some of the learning deficits shown by aged mice.

## Conflict of Interest Statement

The authors declare that the research was conducted in the absence of any commercial or financial relationships that could be construed as a potential conflict of interest.
